# Integrated Analysis of the Altered lncRNA, microRNA, and mRNA Expression in HBV-Positive Hepatocellular Carcinoma

**DOI:** 10.3390/life12050701

**Published:** 2022-05-07

**Authors:** Jingya Yu, Haibin Zhang, Yan Zhang, Xiaolu Zhang

**Affiliations:** 1 Department of Diagnostics, Medical Integration and Practice Center, Cheeloo College of Medicine, Shandong University, Jinan 250012, China; yujingya@sdu.edu.cn (J.Y.); yan.zhang@sdu.edu.cn (Y.Z.); 2 Department of Physiology and Pathophysiology, School of Basic Medical Sciences, Cheeloo College of Medicine, Shandong University, Jinan 250012, China; zhanghbmail@126.com

**Keywords:** lncRNA, microRNA, mRNA, HBV, HCC

## Abstract

Hepatitis B virus (HBV) infection is the most prominent risk factor for developing hepatocellular carcinoma (HCC), which can increase the incidence of HCC by more than 100 times. Accumulated evidence has revealed that non-coding RNAs (ncRNAs) play a regulatory role in various tumors through the long non-coding RNA (lncRNA)–microRNA (miRNA)–mRNA regulation axis. However, the involvement of the ncRNA regulatory network in the progression of HBV infection-induced HCC remains elusive. In the current work, five tumor samples from patients with hepatitis B surface antigen (HBsAg)-positive HCC and three tumor samples from patients with HBsAg-negative HCC were collected for whole-transcriptome sequencing. Between the two groups, 841 lncRNAs, 54 miRNAs, and 1118 mRNAs were identified to be differentially expressed (DE). The Gene Ontology (GO) and Kyoto Encyclopedia of Genes and Genomes (KEGG) analyses indicated that DE genes were mainly involved in cancer-related pathways, including Wnt and MAPK signaling pathways. The Gene Expression Omnibus (GEO) analysis further validated the selected DE mRNAs. The DE lncRNA–miRNA–mRNA network was built to explore the effect of HBV infection on the regulation of ncRNAs in HCC. These findings provide novel insights into the role of HBV infection in the progression of HCC.

## 1. Introduction

Hepatocellular carcinoma (HCC) accounts for approximately 90% of all cases of primary liver malignancy. It is a global health concern associated with an increase in morbidity and mortality worldwide [1]. There are many risk factors for the development of HCC, among which the most notable is infection with hepatitis B virus (HBV), which accounts for about 50% of all HCC cases [2]. Most HCC cases are diagnosed in developing countries, and the regions with high incidence rates coincide with regions where HBV infection is endemic. It is believed that chronic HBV infection elevates the incidence of HCC by more than 100 times [3]. The integration of HBV DNA into the host genome induces both genomic instability and direct insertion mutagenesis in various cancer-related genes. HBV has been found to promote the initiation and progression of HCC depending on the HBV genotype, mutation status, and integration [4]. Moreover, the inflammatory response induced by the expression of the HBV protein also participates in dysregulation of signaling pathways. However, it remains unclear how HBV manipulates the transcriptome and affects the regulatory network in HCC.

Since only about 2–3% of the human transcriptome is protein coding, non-coding RNAs (ncRNAs) have long been described as “genomic noise”. However, in recent years, accumulated studies have revealed the regulatory role of ncRNAs in physiological processes and diseases. MicroRNA (miRNA) is one group of ncRNAs approximately 22 nucleotides long that can target complementary mRNA and result in translation repression or mRNA degradation. Long non-coding RNA (lncRNA) refers to a class of ncRNAs with a length of more than 200 nucleotides. Its regulatory role in miRNA–mRNA binding has been identified [5]. lncRNAs decrease miRNA-induced gene silencing by competitively binding to miRNA response elements as competitive endogenous RNA (ceRNA) [6].

It was found that multiple lncRNAs, such as HULC, HOTAIR, MALAT1, MEG-3, and H19, are closely involved in the tumorigenesis and metastasis of HCC. They also serve as indicators of the HCC diagnosis and prognosis [7]. Recent studies have demonstrated that HBV infection is involved in transcriptome regulation, especially for ncRNAs [8]. HBV DNA integration creates a novel hybrid RNA called HBx-LINE1, which functions as a tumor-promoting lncRNA. lncRNA-HBx-LINE1 can promote HCC by activating Wnt and β-catenin signaling and silencing miR-122 [9,10]. However, the effect of HBV on the function and regulatory network of ncRNAs at the whole-genome expression level remains unclear.

The current study aimed to compare the whole-genome expression landscapes in HCC with or without HBV infection, identify differentially expressed (DE) RNAs related to HBV infection, and demonstrate the HBV-related lncRNA–miRNA–mRNA regulatory network at the whole-genome expression level. High-throughput next-generation sequencing was performed to identify the expression profiles of lncRNAs, miRNAs, and mRNAs in HCC tumors with or without HBV infection. In addition, a systematic bioinformatic analysis of Gene Ontology (GO) and Kyoto Encyclopedia of Genes and Genomes (KEGG) pathways were performed to elucidate the functional annotation of DE genes. The mRNA sequencing data in HBV-positive and HBV-negative HCC samples from Gene Expression Omnibus (GEO) were analyzed to validate the sequencing results.

## 2. Materials and Methods

### 2.1. Patients with HCC 

Eight patients with newly diagnosed histologically confirmed HCC were recruited from Shandong University affiliated Cheeloo Hospital, China. Tumor specimens were obtained from patients who underwent surgical treatment. The presence of tumor cells within collected specimens was verified and samples were frozen at −80 °C until use. The patients were divided into 2 groups by presence of hepatitis B surface antigen (HBsAg), and 5 patients in the HBV-positive group were HBsAg positive and 3 patients in the HBV-negative group were HBsAg negative. The study was approved by the Ethics Committee of Shandong University, China, with an ethics code SDULCLL2021-1-27. Written informed consent was obtained from all patients. 

### 2.2. DNA Extraction and Sanger Sequencing of the HBV Genotype 

Genomic DNA from HCC tumors was extracted using DNA extraction kits (TIANGEN, Beijing, China), and analyzed for the HBV genotype by Sanger sequencing. The S gene of HBV was amplified and sequenced. The primer pair for the HBV S gene amplification was: 5′-CCTGCTGGTGGCTCCAGTTC-3′ (forward) and 5′-GGGTTGCGTCAGCAAACACTT-3′ (reverse). The sequencing primer was 5′-GCAACGGGGTAAAGGTTCA-3′. The results were compared with HBV genotype A-H S gene sequence by BLAST.

### 2.3. lncRNA and mRNA Sequencing 

Total tissue RNA was extracted with the TRIzol reagent kit (Life Technologies, Cat. # 15596-018, Carlsbad, CA, USA). The quality of the total RNA was checked first, using the NanoPhotometer^®^ spectrophotometer (IMPLEN, Westlake Village, CA, USA) with 1% agarose gels. The first strand cDNA was synthesized using 3 μg RNA by M-MuLV reverse transcriptase (RNase H) and random hexamer primer. After PCR, the products were purified by AMPure XP system, and the sequencing library was generated by NEBNext^®^ Ultra^TM^ RNA Library Prep Kit. The library products were sequenced on llumina HiSeq 4000 (Illumina, San Diego, CA, USA). The clean reads without ploy-A/T/C/G and adapter and low-quality reads were used to calculate the FPKM of each gene. The sequencing was performed by Novogene Science and Technology Co., Ltd. (Beijing, China).

### 2.4. Small RNA Sequencing 

For each sample, 3 μg RNA was used for sequencing library building. The libraries were generated using NEBNext^®^Multiplex Small RNA Library Prep Set for Illumina^®^ (NEB, Ipswich, MA, USA). PCR products obtained using LongAmp Taq were purified and subsequently sequenced on Illumina HiSeq 2500 by Novogene Science and Technology Co., Ltd. (Beijing, China). 

### 2.5. lncRNA–miRNA–mRNA Network Analysis

The target genes of miRNA (mRNAs and lncRNAs) were predicted using miRanda and miRBase20.0. The DE mRNAs, miRNAs, and lncRNAs were analyzed by the DESeq R package (1.8.3) using transcriptome sequencing data. After the integrated analysis of DE mRNAs, miRNAs, and lncRNAs and the results obtained from miRanda and miRBase, a global network of DE lncRNAs–DE miRNAs–DE mRNAs was constructed and subsequently visualized by Cytoscape. Available online: https://cytoscape.org/ (accessed on 30 March 2022). 

### 2.6. Statistical Analysis 

GO and KEGG pathway analyses were performed for the statistical enrichment of the DE genes in transcriptome sequencing. The GEO dataset was analyzed for mRNA differential expression between the HBV-positive group and the HBV-negative group. GEO, Available online: https://www.ncbi.nlm.nih.gov/geo/ (accessed on 30 March 2022). The data were collected and shown as mean ± standard deviation (SD). Statistical analysis was performed by Mann–Whitney U test. *p* < 0.05 was considered to be statistically different.

## 3. Results

### 3.1. Profiles of Differentially Expressed lncRNAs, miRNAs, and mRNAs

In the present study, five HBV-positive HCC tumor specimens and three HBV-negative HCC tumor specimens were collected. Sanger sequencing was performed in DNA from the eight HCC samples to detect the presence of HBV DNA and HBV genotype. The sequencing in three HBV-negative samples failed on account of the absence of HBV DNA. The HBV DNA amplification and Sanger sequencing succeeded in the HBV-positive group, and the results showed HBV genotype C in all the five HBV-positive samples. 

**Figure 1:**
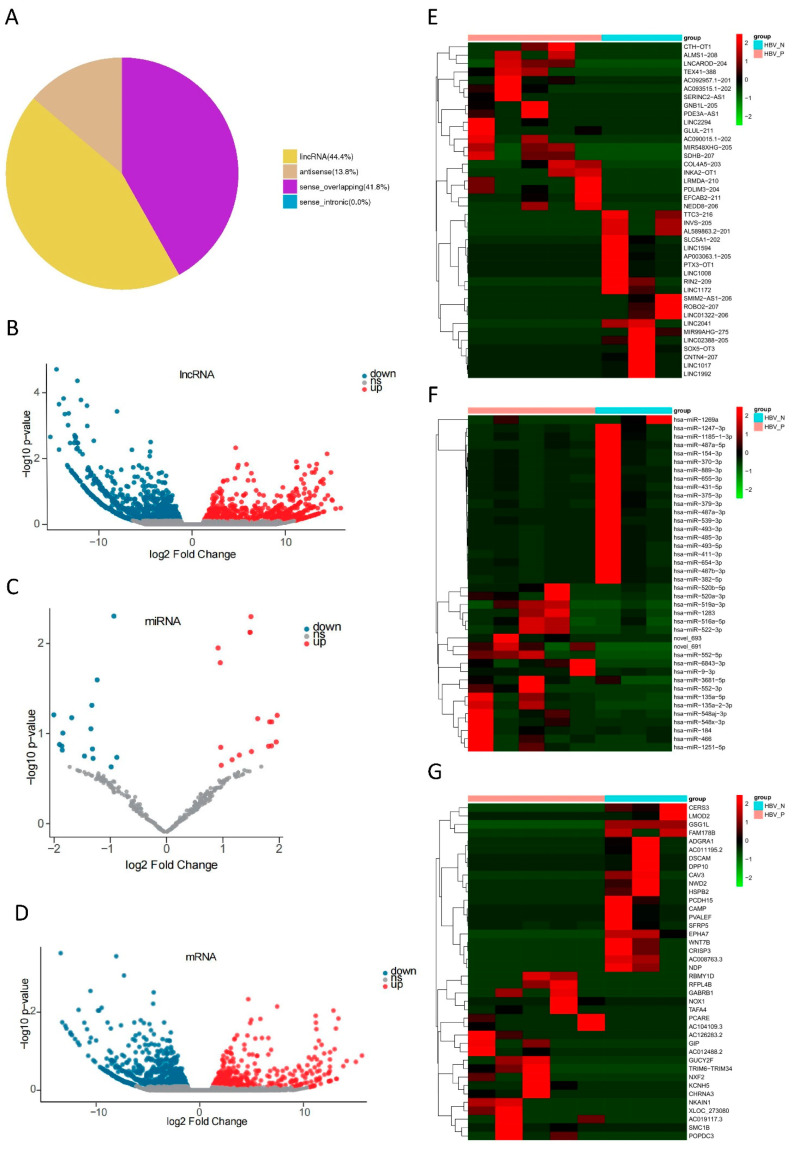
Profiles of differentially expressed (DE) lncRNAs, miRNAs, and mRNAs. (**A**) Pie chart of novel lncRNA classification. Volcano plot of DE lncRNAs (**B**), DE miRNAs (**C**), and DE mRNAs (**D**). Volcano plots show the expression variation of lncRNAs, miRNAs, or mRNAs between the two groups. Each lncRNA, miRNA, or mRNA is represented by a dot, with red dots indicating up-regulation and blue dots indicating down-regulation. The grey dots in the center area indicate no significant difference (*p* < 0.05). Heatmap of fold changes in lncRNAs (**E**), miRNAs (**F**), and mRNAs (**G**). The heatmaps show the hierarchical clustering of altered lncRNAs, miRNAs, or mRNAs between HBV-positive group and HBV-negative group. Red represents up-regulation, and green represents down-regulation.

The eight samples were sent for high-throughput next-generation sequencing. After analyzing the data, a total of 109,862 lncRNAs, 1496 miRNAs, and 83,725 mRNAs were detected from all eight samples. Based on the location to protein-coding genes, lncRNAs can be classified into intergenic lncRNA (lincRNA), sense-intronic lncRNA, sense-overlapping lncRNA, and antisense lncRNA. A total of 6514 novel lncRNAs were identified, among which 44.4% were lincRNAs, 13.8% were antisense lncRNAs, and 41.8% were sense-overlapping lncRNAs (Figure 1A). Compared to the HBV-negative group, expression profiles showed that 712 lncRNAs, 33 miRNAs, and 406 mRNAs were significantly up-regulated, while 129 lncRNAs, 21 miRNAs, and 712 mRNAs were significantly down-regulated (*p* < 0.05) in the HBV-positive group (Figure 1B–D). The heatmaps of DE lncRNAs, DE mRNAs, and DE miRNAs are shown in Figure 1E–G, where two HBV groups are clearly distinguished. In the HBV-positive group, the most up-regulated lncRNAs included CTH-OT1, ALMS1-208, and LNCAROD-204, and the most down-regulated lncRNAs involved LINC1992, LINC1017, and CNTN4-207. The most up-regulated miRNAs included miR-1251-5p, miR-466, and miR-184, and the most down-regulated miRNAs involved miR-1269a, miR-1247-3p, and miR-1185-1-3p. The most up-regulated mRNAs included RBMY1D, RFPL4B, and GABRB1, and the most down-regulated mRNAs involved CERS3, LMOD2, and GSG1L. These findings indicate that HBV infection contributes significantly to the expression landscape changes in HCC.

### 3.2. Differentially Expressed mRNA Validation by GEO Analysis

**Figure 2:**
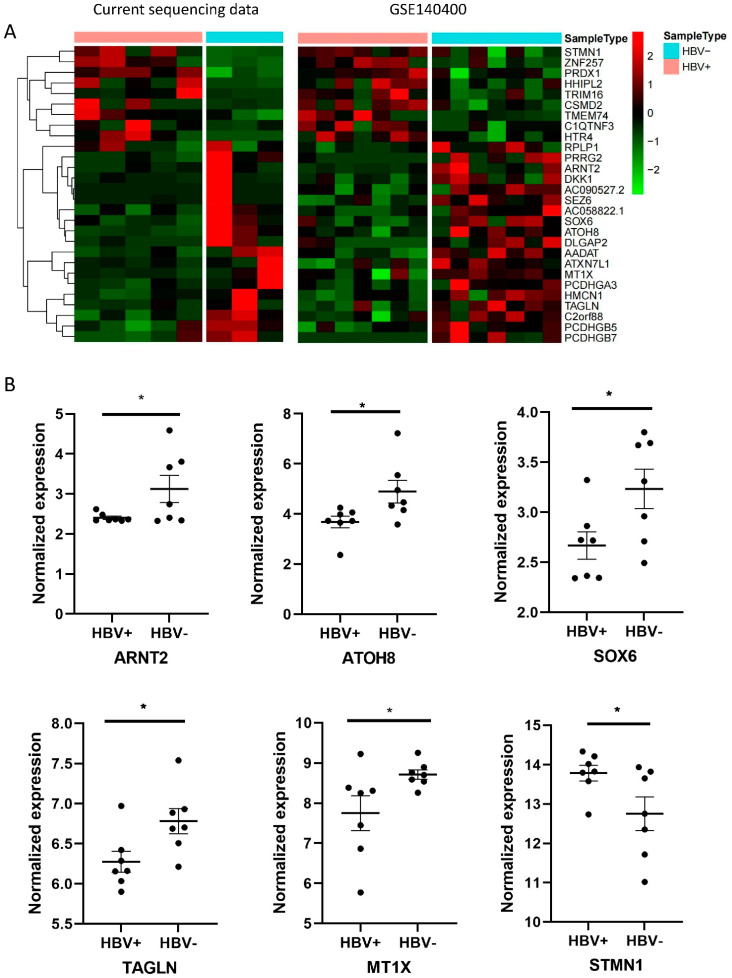
DE mRNA analysis in current sequencing and GSE140400 series indicated 31 common genes. (**A**) Heatmap of 31 common DE mRNAs in current sequencing and GSE140400 series. The heatmaps show the hierarchical clustering of altered mRNAs between the HBV-positive group and HBV-negative group. (**B**) The mRNA expression of ARNT2, ATOH8, MT1X, SOX6, TAGLN, and STMN1 in GSE140400 series was compared between HBV-positive group and HBV-negative group. The mRNA expression of selected gene in each sample is represented by a black dot. * *p* < 0.05.

Sequencing data with HBV infection characteristics were selected from the GEO dataset. In the GSE140400 series, mRNA sequencing was performed in seven HBV-negative and seven HBV-positive HCC samples. Comparing the DE mRNA between the GSE140400 series and the current study’s data, 31 common genes were obtained (Figure 2A). The expression of the selected DE genes in GSE140400, ARNT2, ATOH8, MT1X, SOX6, TAGLN, and STMN1 is presented in Figure 2B. In the GSE140400 HBV-positive group, ARNT2, ATOH8, MT1X, SOX6, and TAGLN were down-regulated, while STMN1 was up-regulated.

### 3.3. Bioinformatic Analysis of Target mRNAs of DE lncRNAs 

**Figure 3:**
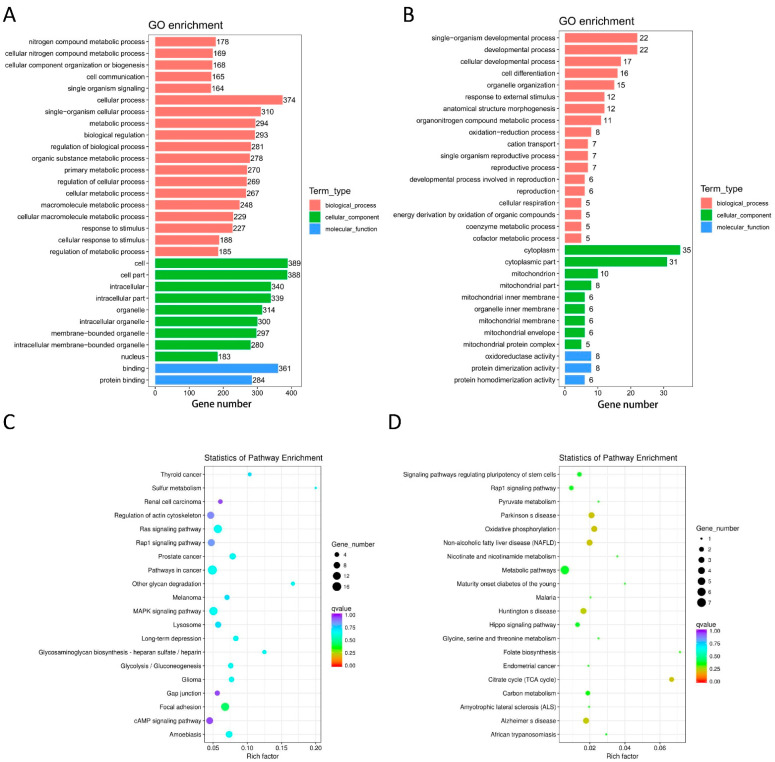
Functional annotation of DE lncRNAs. Bar graphs of GO enrichment analysis (**A**,**B**) and dot plots of KEGG enrichment analysis (**C**,**D**). (**A**) MF, BP, and CC terms of the down-regulated lncRNA-adjacent coding genes. (**B**) MF, BP, and CC terms of the up-regulated lncRNA-adjacent coding genes. (**C**) KEGG enrichment analysis of down-regulated lncRNA-adjacent coding genes. (**D**) KEGG enrichment analysis of up-regulated lncRNA-adjacent coding genes.

The target mRNA of a lncRNA can be predicted by co-location analysis. The GO and KEGG enrichment analyses of the co-located mRNA of DE lncRNA were performed to study the gene function. The GO terms covered three domains: biological processes (BP), cellular components (CC), and molecular functions (MF) in the GO enrichment analysis. The top 30 enrichments are presented in Figure 3A,B. For DE lncRNAs, the most enriched GO terms were metabolic process (GO ID: GO:0008152; type: BP), cellular respiration (GO ID: GO:0045333; type: BP), cell (GO ID: GO:0005623; type: CC), mitochondrion (GO ID: GO:0005739; type: CC), binding (GO ID: GO:0005488; type: MF), and oxidoreductase activity (GO ID: GO:0016491; type: MF).

The top 20 pathways from the KEGG analysis are presented in Figure 3C,D, which correspond to the main pathways of DE lncRNAs, such as Rap1, Ras, MAPK signaling pathways, citrate cycle (TCA cycle), and oxidative phosphorylation.

### 3.4. Bioinformatic Analysis of DE mRNAs

**Figure 4:**
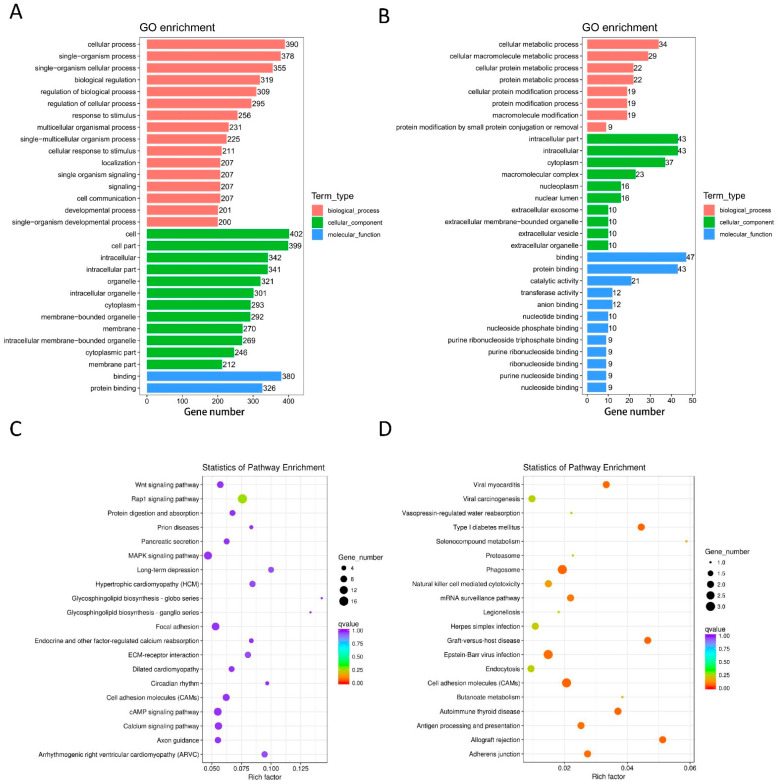
Functional annotation of DE mRNAs. Bar graphs of GO enrichment analysis (**A**,**B**) and dot plots of KEGG enrichment analysis (**C**,**D**). (**A**) MF, BP, and CC terms of the down-regulated mRNAs. (**B**) MF, BP, and CC terms of the up-regulated mRNAs. (**C**) KEGG enrichment analysis of down-regulated mRNAs. (**D**) KEGG enrichment analysis of up-regulated mRNAs.

For DE mRNAs, the most enriched GO terms were single-organism process (GO ID: GO:0044699; type: BP), protein modification by small protein conjugation or removal (GO ID: GO:0070647; type: BP), cell (GO ID: GO:0005623; type: CC), nucleoplasm (GO ID: GO:0005654; type: CC), binding (GO ID: GO:0005488; type: MF), and protein binding (GO ID: GO:0005515; type: MF) (Figure 4A,B). 

The top 20 pathways from the KEGG analysis are presented in Figure 4C,D, which correspond to the main pathways of DE mRNAs, such as the Wnt, Rap1, cAMP, and MAPK signaling pathways, as well as hypertrophic cardiomyopathy (HCM).

### 3.5. Bioinformatic Analysis of Target mRNAs of DE miRNA 

**Figure 5:**
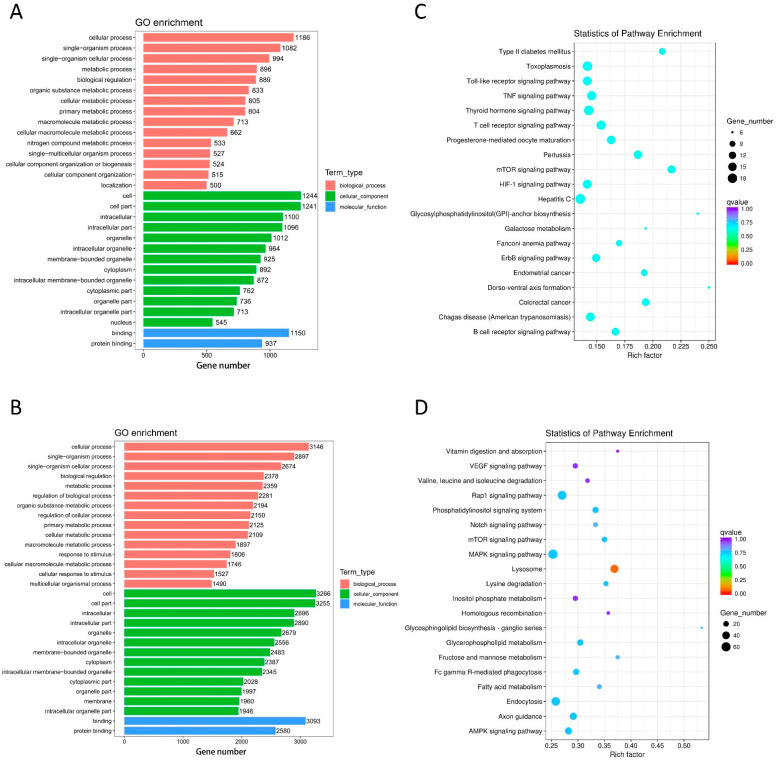
Functional annotation of target mRNAs of DE miRNAs. Bar graphs of GO enrichment analysis (**A**,**B**) and dot plots of KEGG enrichment analysis (**C**,**D**). (**A**) MF, BP, and CC terms of the target mRNAs of down-regulated miRNAs. (**B**) MF, BP, and CC terms of the target mRNAs of up-regulated miRNAs. (**C**) KEGG enrichment analysis of the target mRNAs of down-regulated miRNAs. (**D**) KEGG enrichment analysis of the target mRNAs of up-regulated miRNAs.

The GO and KEGG enrichment analyses of DE miRNA-targeted mRNAs were performed to study the gene function. The top 30 enrichments are presented in Figure 5. For DE miRNAs, the most enriched GO terms were cell (GO ID: GO:0005623; type: CC), cell part (GO ID: GO:0044464; type: CC), binding (GO ID: GO:0005488; type: MF), protein binding (GO ID: GO:0005515; type: MF), cellular process (GO ID: GO:0009987; type: BP), and single-organism process (GO ID: GO:0044699; type: BP) (Figure 5A,B). 

The top 20 pathways from the KEGG analysis are presented in Figure 5C,D, which correspond to the main pathways of DE miRNAs, such as the MAPK, mTOR, and ERBB signaling pathways.

### 3.6. lncRNA–miRNA–mRNA Regulatory Network Analysis 

**Figure 6:**
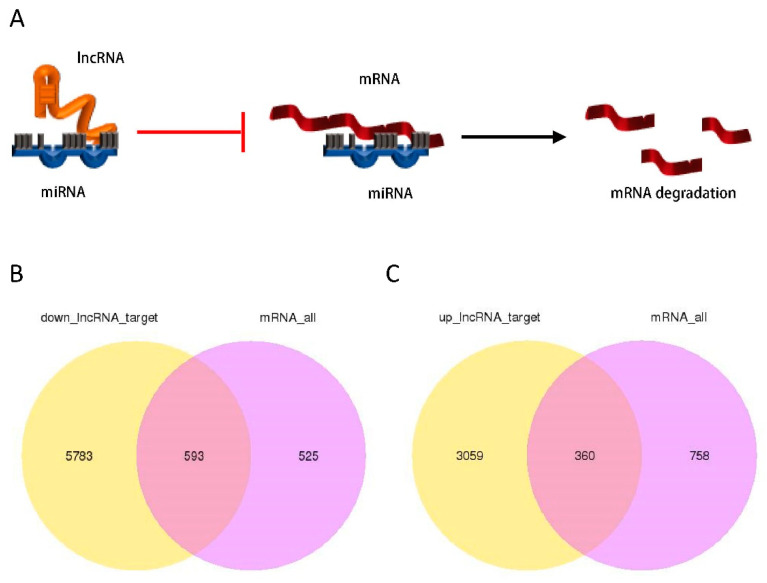
lncRNA–miRNA–mRNA network analysis. (**A**) lncRNA inhibits miRNA–mRNA binding as a competitive endogenous RNA (ceRNA) and therefore inhibits mRNA degradation. (**B**) Venn diagram of the target mRNAs of down-regulated lncRNAs and DE mRNAs. (**C**) Venn diagram of the target mRNAs of up-regulated lncRNAs and DE mRNAs. In Venn diagram, yellow indicates targeted mRNAs of DE lncRNAs, and purple for DE mRNAs.

The mechanism of how lncRNA regulates mRNA expression through miRNA is sponging. By binding to 3′UTR of the complementary mRNA, miRNA promotes mRNA degradation and regulates mRNA expression at the post-transcriptional level. Complementary lncRNA can bind to miRNA as a competitive endogenous RNA (ceRNA) and subsequently inhibit miRNA–mRNA binding. Eventually, lncRNA up-regulates the expression of downstream mRNA by sponging (Figure 6A). To further explore the regulatory functions of lncRNAs in HBV-positive HCC, a lncRNA–miRNA–mRNA network was constructed based on whole-transcriptome sequencing. Expression profiles of the lncRNAs showed that there were 6376 target mRNAs of down-regulated lncRNAs, while 593 mRNAs were shared with DE mRNA. On the other hand, 360 of the 3419 target mRNAs of up-regulated lncRNAs were shared with DE mRNA (Figure 6B,C).

**Figure 7:**
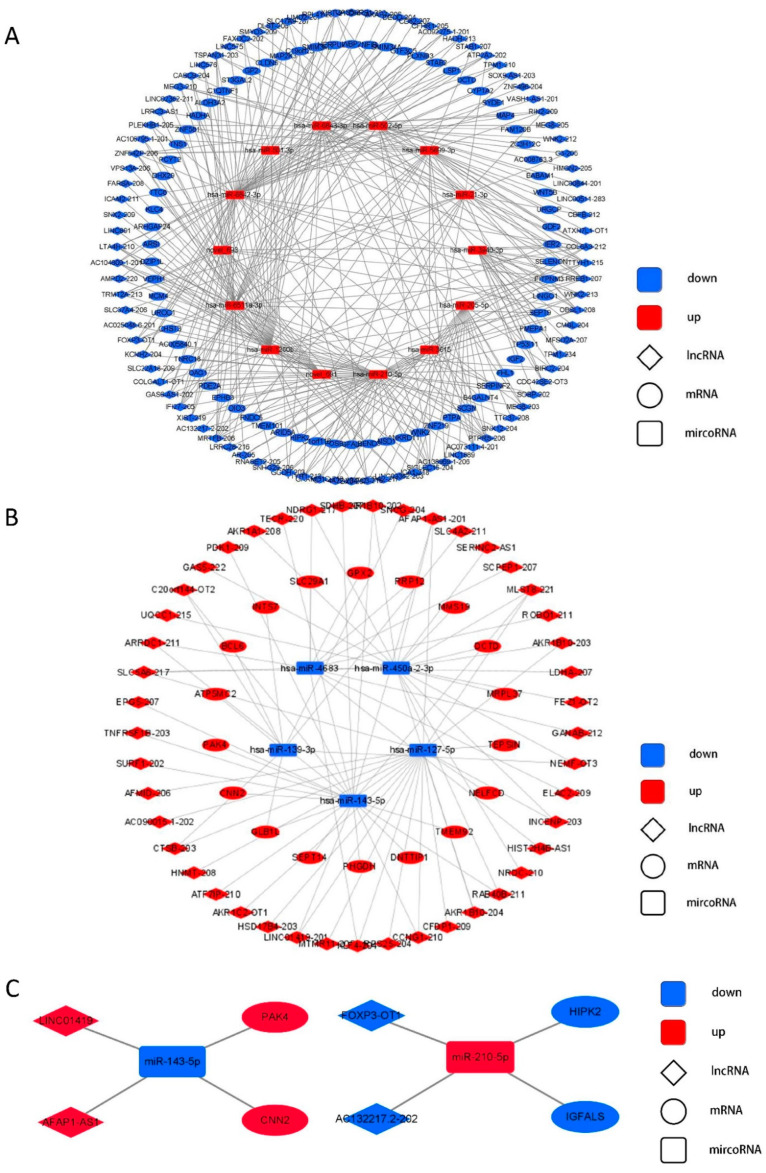
The ‘down-up-down’ and ‘up-down-up’ lncRNA–miRNA–mRNA network. (**A**) Network of down-regulated lncRNA-up-regulated miRNA-down-regulated mRNA. (**B**) Network of up-regulated lncRNA-down-regulated miRNA-up-regulated mRNA. (**C**) Selected ‘down-up-down’ and ‘up-down-up’ lncRNA–miRNA–mRNA axes. The rhombuses represent lncRNAs, rectangles represent miRNAs, and circles represent mRNAs. Red means up-regulation and blue means down-regulation.

To construct the lncRNA–miRNA–mRNA network in HBV-positive HCC, DE lncRNA-targeted DE miRNAs and DE miRNA-targeted DE mRNAs were analyzed. DE RNAs were divided into two groups depending on whether they were up-regulated or down-regulated. For the group ‘down-up-down’, 92 down-regulated lncRNAs, 14 up-regulated miRNAs, and 72 down-regulated mRNAs were selected. For the group ‘up-down-up’, 45 up-regulated lncRNAs, 5 down-regulated miRNAs, and 18 up-regulated mRNAs were selected (FPKM ≥ 1.0, fold change ≥ 1.0, *p* < 0.05). Each lncRNA can bind to multiple miRNAs, and each miRNA can target multiple mRNAs. The DE lncRNA–DE miRNA–DE mRNA network is presented in the form of the ‘down-up-down’ and ‘up-down-up’ groups in Figure 7A,B, respectively. The selected ‘up-down-up’ axis AFAP1-AS1/LINC01419-miR-143-5p-PAK4/CNN2 and ‘down-up-down’ axis AC132217.2-202/FOXP3-OT1-miR-210-5p-HIPK2/IGFALS are presented in Figure 7C.

## 4. Discussion

It has been demonstrated that HBV is the most prominent risk factor for the development of HCC. Chronic HBV infection can lead to hepatitis, liver cirrhosis, and finally, liver cancer. Studies have revealed that the mechanisms of HBV in tumorigenesis and progression of HCC can be either direct or indirect. The viral regulatory protein HBx dysregulates transcription and cell proliferation, and the integration of HBV DNA into the human genome can cause genomic instability and increase the risk of oncogene mutation. However, the association between HBV infection and oncogene mutations, such as those in the *CTNNB1* mutation and *TERT* promoter, was not significant. Further efforts are necessary to explore the detailed mechanism of HBV in HCC.

Recently, the role of HBV in the regulation of ncRNAs has been characterized. HBV can modulate the expression of ncRNA, which can participate in the regulation of HCC-relevant pathways, such as JAK/STAT, TP53, Wnt/β-catenin, and PI3K/MAPK pathways. Studies have demonstrated that a few lncRNAs and miRNAs are dysregulated by HBV in HBV-HCC. HBx increases the expression of oncogenic lncRNA, such as HULC, MALAT1, and UCA1 [11,12,13,14]. Results in the current study also showed an up-regulated expression of HULC in HBV-HCC, which aligns with the results of previous studies. 

MiR-205-5p and miR-210-5p, also reported as cancer-promoting miRNAs in lung cancer and osteosarcoma, are also significantly up-regulated in HBV-HCC, as demonstrated in the current study [15,16].

The GO and KEGG analyses of DE lncRNA co-located mRNAs, DE mRNAs, and the DE miRNA target mRNAs altogether revealed the role of HBV in the overall gene function. According to the GO analysis, DE RNAs were primarily involved in the metabolic process and protein binding in HBV-HCC. The KEGG analysis showed that the abovementioned mRNAs were mainly involved in the Wnt, MAPK, mTOR, Ras, and Rap1 signaling pathways. Ras proteins are small GTPases that regulate multiple cellular processes, and Rap1 is another GTPase similar to Ras that also mediates Ras functions. Ras and Rap1 cooperate to stimulate ERK signaling and promote malignancy. The MAPK and AKT/mTOR pathways are generally disrupted in HCC, while the Wnt pathway, a canonical cancer-related pathway, is up-regulated in many cases of early HCC [17,18,19]. It has been reported that HBx-related ncRNAs are involved in the regulation of HCC-related pathways. Typically, miRNAs up-regulated by HBx inhibit tumor suppressors and miRNAs down-regulated by HBx fail to modulate oncogenic proteins. In the AKT/mTOR pathway, HBx inhibits the tumor suppressor PTEN by up-regulating miR-21 [20]. miR-21 also enhances Wnt signaling while simultaneously inhibiting PDCD4 expression, thus contributing to the suppression of E-cadherin [21]. Moreover, it has been reported that miR-21 is also up-regulated in HCC [22]. The current study showed that the expression of miR-21 was significantly up-regulated in the HBV-positive HCC group, which corresponds to the results of previous studies.

An intergroup analysis of the lncRNA–miRNA–mRNA network revealed several lncRNA–miRNA–mRNA axes that might be affected by HBV. P21-activated kinase 4 (PAK4) has been demonstrated to function as an oncogenic protein in various cancers, including HCC [23]. Another protein, calponin 2 (CNN2), has been reported to promote cell proliferation and migration in HCC [24]. These two genes were up-regulated in the HBV-positive HCC group, and both of them were the target of miR-143-5p, which was down-regulated in the HBV-positive HCC group. miR-143-5p is an antitumor miRNA that plays a prominent role in multiple cancers, including breast cancer and gastric cancer, but its role in HCC remains unclear [25,26]. Fourteen DE lncRNAs, including AFAP1-AS1 and LINC01419, were found to competitively bind to miR-143-5p. AFAP1-AS1 promotes the development of various cancers and indicates a poor prognosis for HCC, while LINC01419 promotes cell proliferation and metastasis in HCC [27,28]. Taken together, AFAP1-AS1/LINC01419-miR-143-5p-PAK4/CNN2 represents a potential tumor-promoting up-down-up axis.

In addition to the up-down-up axes, the down-up-down axes were also identified. Serine/threonine homeodomain-interacting protein kinase 2 (HIPK2) and insulin-like growth factor binding protein, acid labile subunit (IGFALS) were down-regulated in HBV-positive HCC, and both were target mRNAs of miR-210-5p. HIPK2 promotes the degradation of HIF-1α, suppressing tumor growth and progression of HCC [29]. IGFALS has been reported to function as a tumor suppressor in HCC [30]. miR-210-5p was up-regulated in HBV-positive HCC. Although its role in HCC remains unclear, studies have reported that miR-210-5p functions as a cancer-promoting miRNA in osteosarcoma [16]. Twenty-seven down-regulated lncRNAs, including AC132217.2-202 and FOXP3-OT1, had the potential to bind to miR-210-5p in HBV-positive HCC. 

Previous studies have reported that in developing countries, HCC, in most cases, overlaps with HBV infection. The current study showed consistent results; out of more than 70 HCC samples collected in total, only 3 HBV-negative HCC samples were obtained. To confirm the sequencing results, sequencing data with HBV infection characteristics were screened in the GEO dataset, and the GSE140400 series was selected for the validation. The GSE140400 series contained mRNA sequencing data from seven HBV-positive and seven HBV-negative HCC samples, which were analyzed for DE mRNA. In the end, 31 common significant DE genes shared with the current study were obtained, which validated the current sequencing results. 

In summary, according to the current study’s findings, HBV could affect HCC in various aspects. Based on the canonical analysis, including the GO and KEGG pathway analyses, HBV appears to be involved in the regulation of cancer-related pathways. A comprehensive analysis of lncRNAs, miRNAs, and mRNAs revealed that HBV may affect the lncRNA–miRNA–mRNA regulatory network in HCC.

## Data Availability

The data presented in this study are available on request from the corresponding author. The data are not publicly available due to privacy.
